# Small intestinal organoids as a model to study interactions of *C. suis* with its porcine host

**DOI:** 10.3389/fmicb.2026.1818217

**Published:** 2026-04-29

**Authors:** Margaux Verhaeghe, Jan Gettemans, Daisy Vanrompay, Bert Devriendt

**Affiliations:** 1 Department of Animal Sciences and Aquatic Ecology, Faculty of Bioscience Engineering, Ghent University, Ghent, Belgium; 2 Department of Biomolecular Medicine, Faculty of Medicine and Health Sciences, Ghent University, Ghent, Belgium; 3 Department of Translational Physiology, Infectiology, and Public Health, Faculty of Veterinary Medicine, Ghent University, Merelbeke, Belgium

**Keywords:** *C. suis*, enteroid-derived monolayers, gut epithelium, jejunum, porcine enteroids

## Abstract

*Chlamydia suis* is an obligate intracellular bacterium endemic in pig populations and is detected in the gastrointestinal tract, suggesting that the intestine may be an important site of chlamydial colonization. Despite this, intestinal chlamydial infections remain poorly understood, largely due to the lack of models that accurately mimic the interaction of *C. suis* with the gut epithelium. The aim of this study was to evaluate whether porcine jejunum-derived enteroids constitute a suitable *in vitro* model to investigate intestinal infection by *C. suis* and to compare infection dynamics with the closely related human pathogen *Chlamydia trachomatis*. Porcine enteroid monolayers were exposed to *C. suis* and *C. trachomatis*, and bacterial uptake, inclusion formation, and replication were assessed using microscopy-based and molecular approaches. Both *C. suis* and *C. trachomatis* efficiently attached to primary intestinal epithelial cells and formed intracellular inclusions, indicating successful bacterial uptake and early intracellular survival. Infection levels increased in a dose-dependent manner, confirming that enteroid-derived monolayers are suitable for studying early host–pathogen interactions. Furthermore, treatment with tetracycline reduced *C. suis* inclusion formation and extracellular bacterial release, thereby functionally confirming that an active infection had been established and that the model responded as expected to antibiotic treatment. However, unlike the McCoy cell model which supports productive bacterial replication, enteroid cultures did not exhibit a significant increase in intracellular bacterial load over time. Despite this limited replication in enteroids, chlamydial DNA accumulated in the culture supernatants, suggesting extracellular release of bacterial material. In conclusion, porcine intestinal epithelial cells permit chlamydial attachment, internalization, and early inclusion formation. However, under the tested conditions, efficient completion of the chlamydial developmental cycle was not observed. Nevertheless, porcine enteroid-derived cultures represent a physiologically relevant *in vitro* platform to investigate the early stages of intestinal chlamydial infection and provide a valuable system for studying host–pathogen interactions at the intestinal interface.

## 1 Introduction

*Chlamydia (C.) suis* is an obligate intracellular bacterium endemic in pig populations worldwide and represents the most prevalent chlamydial species infecting swine. In pigs, *C. suis* infection is associated with a broad spectrum of clinical manifestations, including conjunctivitis, respiratory disease, enteritis, diarrhea, reproductive disorders, and polyarthritis, while subclinical infections are also common (Borel et al., 2012). These disease manifestations adversely affect animal welfare, growth performance, and reproductive efficiency, thereby resulting in economic losses in pig production systems (De Puysseleyr et al., 2014a, 2014b). *C. suis* is detected in respiratory and reproductive samples and in conjunctival swabs, but it is also identified in rectal swabs or fecal samples (Schautteet et al., 2013; De Puysseleyr et al., 2014a, 2017a). Several studies have reported the presence of *C. suis* in both the small and large intestinal tract of pigs suggesting that the intestinal tract represents a site of chlamydial colonization (Yeruva et al., 2013; Hoffmann et al., 2015; Wanninger et al., 2016; Li et al., 2017; Verhaeghe et al., 2026).

*C. suis* is highly related to the human pathogen *C. trachomatis* (Everett et al., 1999). *C.trachomatis* is primarily associated with urogenital and ocular infections. However, like observed in pigs, human intestinal colonization has also been described for *C. trachomatis* and *C. trachomatis* has been detected in rectal samples from women without a history of anal sexual intercourse, suggesting autoinoculation or migration from the female genital tract (Zhong, 2018). The biological consequences of intestinal colonization, particularly with respect to persistence, transmission, and reinfection, remain poorly understood (Zhong, 2021; Tian et al., 2024). Together, observations in pigs and humans thus underscore the importance of the intestinal tract as a relevant but underexplored site of chlamydial infection and highlight the need for physiologically relevant intestinal models to study chlamydial host-pathogen interactions and pathogenesis.

Members of the family *Chlamydiaceae* exhibit a characteristic biphasic developmental cycle that alternates between the infectious elementary body (EB) and the metabolically active reticulate body (RB). Following internalization into host cells, EBs differentiate into RBs within a membrane-bound intracellular vacuole, referred to as an inclusion, which is derived from the host cell membrane and serves as the replicative niche of the bacterium. Within these inclusions, EBs differentiate into RBs during the early stage of infection (approximately 6-8 h post-infection (p.i.)). RBs subsequently replicate by binary fission, leading to expansion of the inclusion. During the late stage of infection (approximately 48 h p.i.), RBs asynchronously re-differentiate into EBs that are released from the host cell to infect neighboring cells. Depending on the *Chlamydia* species and host cell type, a complete developmental cycle typically spans approximately 48–72 h (Matsumoto and Manire, 1970; Moulder, 1991; Elwell et al., 2016).

Traditionally, the isolation of *C. suis* (Suchland et al., 2003; Sachse et al., 2009; De Puysseleyr et al., 2014a, 2014b, 2017a) and studies on *C. suis* bacterium-host cell interactions (Schiller et al., 2004; De Puysseleyr et al., 2017b; Marti et al., 2017; De Puysseleyr et al., 2021) have mainly relied on immortalized cell lines such as Vero and McCoy cells, due to their high susceptibility and ease of culture. While cell lines are suitable for diagnostic purposes, they lack the structural complexity and heterogeneity of the intestinal epithelium. In contrast, enteroids are known to recapitulate several key characteristics of the intestinal epithelium, including epithelial polarization and the capacity to differentiate into multiple intestinal epithelial cell types (van der Hee et al., 2018; Li et al., 2019; Mussard et al., 2023). Enteroids can be maintained as 3D structures embedded in extracellular matrix (ECM) or converted into 2D monolayers on ECM-coated surfaces, enabling direct access to the apical side of the epithelium, which facilitates the study of pathogen-intestinal epithelium interactions (Vermeire et al., 2021).

A recent study employed human intestinal organoids to model enteric *C. trachomatis* infections (Hovhannisyan et al., 2024). However, to our knowledge, infection of primary intestinal epithelial cells with *C. suis* using porcine enteroids has not yet been investigated. Porcine small intestinal enteroids therefore represent a highly relevant *in vitro* model for studying intestinal chlamydial infections, particularly considering the preferential colonization of the small intestine observed in pigs (Guscetti et al., 2009; Verhaeghe et al., 2026).

In this study, we describe a porcine infection model for *C. suis* using jejunum-derived enteroids. Two-dimensional monolayers generated from fragmented enteroid cultures were infected with either the *C. suis* U.S. strain R19 or the S45 reference strain. Given the close phylogenetic relationship between *C. suis* and *C. trachomatis*, and ability of both pathogens to naturally or experimentally infect pigs (Guscetti et al., 2009; De Clercq et al., 2013; Dimond and Hefty, 2021), we compared the infection biology of the two species in porcine enteroids. Previous work has shown that *C. trachomatis* can replicate in human intestinal organoids (Hovhannisyan et al., 2024). Here, we extend these observations to a porcine intestinal epithelial model. This approach allowed us to assess whether porcine jejunal enteroids provide a suitable *in vitro* system for studying infections with *Chlamydia* species.

## 2 Materials and methods

### 2.1 *Chlamydia* strains

*Chlamydia suis* strains R19 (kindly provided by A. Andersen, lowa State University, U.S.) and S45 (ATCC, Manassas, Virginia, US), and *Chlamydia trachomatis* E/Bour (DSMZ, Braunschweig, Germany) were used in this study. The *C. suis* reference strain R19 is tetracycline-susceptible, whereas strain S45 displays a tetracycline-resistant phenotype (Schautteet et al., 2013; Donati et al., 2016).

For bacterial stock preparations, strains were propagated in McCoy cells (mouse fibroblast cell line, CRL-1696, American Type Culture Collection) in Eagle’s Minimum Essential Medium (Thermo Fisher Scientific, Paisley, UK) following standard procedures (Vanrompay et al., 1992). After 6 days of incubation, cells were harvested in culture medium using a sterile cell scraper, sonicated (1 min at 37 kHz) and centrifuged (1100 × *g*, 10 min, 4 °C) to remove cell debris. Bacteria in supernatant were subsequently pelleted by ultracentrifugation at 72,660 × *g* for 45 min at 4 °C. The resulting *Chlamydia* pellets were resuspended in 1 × sucrose-phosphate-glutamate (SPG) buffer, aliquoted, and stored at −80 °C.

### 2.2 Culture of porcine enteroids

Jejunal crypts were isolated from two 3- to 6-week-old piglets as previously described (Vermeire et al., 2021). Crypts were embedded in Cultrex UltiMatrix RGF BME domes (Bio-Techne R&D Systems, Minneapolis, USA) and IntestiCult OGM medium (STEMCELL Technologies) in 24-well plates at 37 °C and 5% CO_2_. The resulting enteroids were passaged every 7–10 days. The domes were washed twice with 500 μL cold sterile PBS (Gibco, Paisley, Scotland). Domes were incubated on ice for 30 min with 500 μL Cell Recovery Solution (Corning, Lasne, Belgium) to release the enteroids. Structures were collected by centrifugation at 200 × *g*, 4 °C for 5 min, resuspended in 800 μL PBS containing 10 μM Y-27632 dihydrochloride (Sigma-Aldrich, Hoeilaart, Belgium), and fragmented by passage through a 21G needle. After centrifugation (200 × *g*, 4 °C, 5 min), pellets were resuspended in 32 μL Cultrex UltiMatrix (supplemented with 2% 10 mM Y-27632 and 16% IntestiCult OGM), plated into pre-warmed 24-well plates, polymerized at 37 °C for 15–30 min, and overlaid with 350 μL IntestiCult OGM. Medium was refreshed every 2–3 days during the 7–10 days of culture.

### 2.3 Enteroid-derived 2D monolayers

Generation of 2D monolayers was performed as described (Vermeire et al., 2021). Wells of 24-well plates, with or without 13 mm glass inserts, were coated with 2.5 μg/cm^2^ mouse Collagen IV (Corning, Lasne, Belgium) for 1 h at room temperature, washed with cold sterile PBS, and air-dried. Gut organoid fragments were seeded on the Collagen IV-coated wells and cultured in IntestiCult OGM at 37 °C and 5% CO₂. Medium was refreshed every 2–3 days. Confluence was typically reached after 6–7 days.

### 2.4 *Chlamydia* infection biology in McCoy cells and enteroid-derived 2D monolayers

In this study, McCoy cells, a widely used cell line for *C. suis* and *C. trachomatis* growth, were used as reference artificial host and strains were thus inoculated in parallel in McCoy cells and in enteroids. Confluent enteroid monolayers or McCoy monolayers were inoculated with *C. suis* S45 or R19, or *C. trachomatis* Bour. Following inoculation, enteroid monolayers were centrifuged at 800 × *g* for 60 min at 37 °C, whereas McCoy monolayers were centrifuged at 1,900 × *g* for 60 min at 37 °C to promote bacterial contact with host cells. Subsequently, inoculated enteroid monolayers were incubated at 37 °C with 5% CO₂ in IntestiCult OGM-based *Chlamydia* culture medium, while inoculated McCoy monolayers were incubated under identical conditions, but in *Chlamydia* culture medium. Inoculations were always performed in duplicate.

Different aspects of chlamydial infection biology were examined, including host attachment, infectivity, and infection kinetics of both *C. suis* and *C. trachomatis*. For host attachment and infectivity, experiments were independently repeated using enteroids derived from another pig, again using McCoy cells as reference artificial host.

Different inoculation doses were used depending on the objective and sensitivity of the respective assays. For the attachment assays, inoculation doses were selected based on preliminary optimization experiments to ensure measurable and comparable signals within the linear detection range of the qPCR assay for each *Chlamydia* species. As *C. suis* and *C. trachomatis* differ in infectivity and replication efficiency in cell culture, slightly different inoculum sizes were required to obtain comparable detectable levels. For infectivity assays, multiple inoculation doses were included to assess dose–response relationships and to ensure that the observed effects were not restricted to a single infection level.

For host attachment assays, inocula of 10^5^ inclusion forming units per milliliter (IFU/mL) *C. suis* R19 and 10^6^ IFU/ml *C. trachomatis* Bour were prepared in duplicate. For each strain, one inoculum was stored at −80 °C for subsequent DNA extraction and species-specific real-time PCRs, while the second inoculum was used to infect enteroid and McCoy monolayers. Following centrifugation of the monolayers, non-attached C*hlamydiae* were removed and stored briefly at −80 °C until DNA extraction and species-specific real-time PCR analysis.

For infectivity assays, monolayers were inoculated with three concentrations of *C. suis* R19 or *C. trachomatis* Bour (10^7^, 10^6^, or 10^5^ IFU/mL). After 2 days of incubation, monolayers were fixed with methanol and stained. Ten random microscopic fields per sample were acquired using a fluorescence microscope (Leica Thunder). The total infected area (μm^2^) and the number of nuclei per field were quantified using QuPath software (version 0.6.0). To account for differences in cell density between fields of view, the FITC-positive area was normalized to the number of nuclei, yielding a normalized FITC-positive area.

For studying infection kinetics, enteroid monolayers and McCoy monolayers were infected in quadruplicate with 10^7^ IFU/mL *C. suis* R19 or 10^7^ IFU/mL *C. trachomatis* Bour. Two monolayers per condition were used for supernatant collection, with culture supernatants harvested at 2 h, 24 h, 48 h, and 72 h post-infection and stored at −80 °C until DNA extraction and species-specific real-time PCR analysis. From the other two monolayers, cells were harvested using a sterile cell scraper, pelleted by centrifugation at 18,000 × *g* for 30 min, resuspended in 200 μL PBS, and stored at −80 °C until DNA extraction species-specific real-time PCR analysis.

### 2.5 Functional validation of infection

For functional validation of the infection and for evaluating the model responsiveness to antibiotic treatment, tetracycline experiments were performed. First, inclusion morphology was compared between *C. suis* S45 (tetracycline-sensitive) and *C. suis* R19 (tetracycline-resistant). Here, enteroid monolayers were inoculated in duplicate with 10^7^ IFU/mL *C. suis* S45 and, in parallel, in duplicate with 10^7^ IFU/mL *C. suis* R19. After 2 days of incubation at 37 °C and 5% CO_2_, monolayers were fixed with methanol, stained, and imaged using a fluorescence microscope (Leica Thunder). The experiment was independently repeated using enteroids derived from a second pig.

In addition, a tetracycline growth inhibition assay was performed. For this purpose, enteroid monolayers were infected in quadruplicate with *C. suis* S45 at an inoculum of 10^9^ IFU/mL. A higher inoculum was used to ensure robust infection and sufficient bacterial load for reliable quantification of growth inhibition. After centrifugation, two monolayer were cultured in standard *Chlamydia* culture medium, whereas the other two monolayers were cultured in medium supplemented with 0.1 mg/mL tetracycline. After 2 days of incubation at 37 °C and 5% CO₂, culture supernatants were collected and stored at −80 °C for DNA extraction and *C. suis* real-time PCR analysis. Monolayers were subsequently fixed with methanol, stained, and imaged using a fluorescence microscope (Leica Thunder). The experiment was independently repeated using enteroids derived from a second pig.

### 2.6 Immunofluorescence staining

Infected monolayers were fixed with methanol for 10 min prior to detection of *C. suis* or *C. trachomatis* using the IMAGEN™ *Chlamydia* Reagent (Oxoid™, Geel, Belgium) (Gibco, Paisley, Scotland). This reagent contains a genus-specific FITC-conjugated monoclonal antibody against *Chlamydia* lipopolysaccharide (LPS). The 24 well plates were incubated in a humid chamber at 37 °C for 45 min. Subsequently, nuclei were stained with DAPI. Cells were washed twice with 2 mL PBS (Gibco, Paisley, Scotland) and twice with 2 mL Aqua BiDest. Glass inserts were removed from the wells, air dried, and placed with cells facing downwards onto a droplet of Mounting Fluid (ThermoFisher Scientific, Hampshire, England) on microscope slides (VWR, Leuven, Belgium). Images were acquired using a fluorescence microscope (Leica Thunder, Diegem, Belgium). Image analysis was performed as described above.

### 2.7 DNA extraction and real-time PCRs

Genomic DNA from McCoy cells or enteroids was extracted using the QIAamp® DNA Mini Kit (Qiagen, Antwerp, Belgium) following the manufacturer’s instructions.

DNA samples containing *C. suis* R19 or S45 were analyzed using a species-specific TaqMan probe-based *C. suis* real-time PCR assay, as previously described (De Puysseleyr et al., 2014b), with one modification: the original inhibition control plasmid was replaced by a Universal Exogenous Internal Positive Control for TaqMan assays (Eurogentec, Belgium). The analytical sensitivity of the assay was 10 copies of 23S rDNA per reaction. Samples with Ct values < 35 were considered positive for *C. suis*. Bacterial loads were estimated by converting Ct values using a standard curve describing the correlation between Ct values and IFU equivalents per mL. The standard curve was generated by performing real-time PCR on a ten-fold dilution series of genomic DNA extracted from *C. suis* S45 with a defined infectious titer (IFU/mL) per reaction. The resulting values therefore represent qPCR-derived estimates of bacterial load rather than direct measurements of viable inclusion-forming units.

DNA samples containing *C. trachomatis* were analyzed using the commercially available PRESTO assay according to the manufacturer’s instructions (Goffin Molecular Technologies, Waalwijk, The Netherlands). Samples with Ct values < 35 were considered positive for *C. trachomatis*. Bacterial loads were estimated by converting Ct values using a standard curve generated from a ten-fold dilution series of genomic DNA from *C. trachomatis* Bour with a defined IFU/mL concentration per reaction. The resulting values therefore represent qPCR-derived estimates of bacterial load rather than direct measurements of viable inclusion-forming units.

### 2.8 Statistical analysis

FITC-positive areas across the different inoculation doses were compared using one-way Welch’s ANOVA, as the data were heteroscedastic, followed by Dunnett’s multiple-comparisons *post hoc* test. *p*-values < 0.05 were considered statistically significant. Statistical significance is indicated as follows: *p* > 0.05 (ns), *p* < 0.05 (*), *p* < 0.01 (**), *p* < 0.001 (***), and *p* < 0.0001 (****). All statistical analyses were performed using GraphPad Prism version 8.0.1.

## 3 Results

### 3.1 *Chlamydia* attaches to primary intestinal epithelial cells

**Figure 1:**
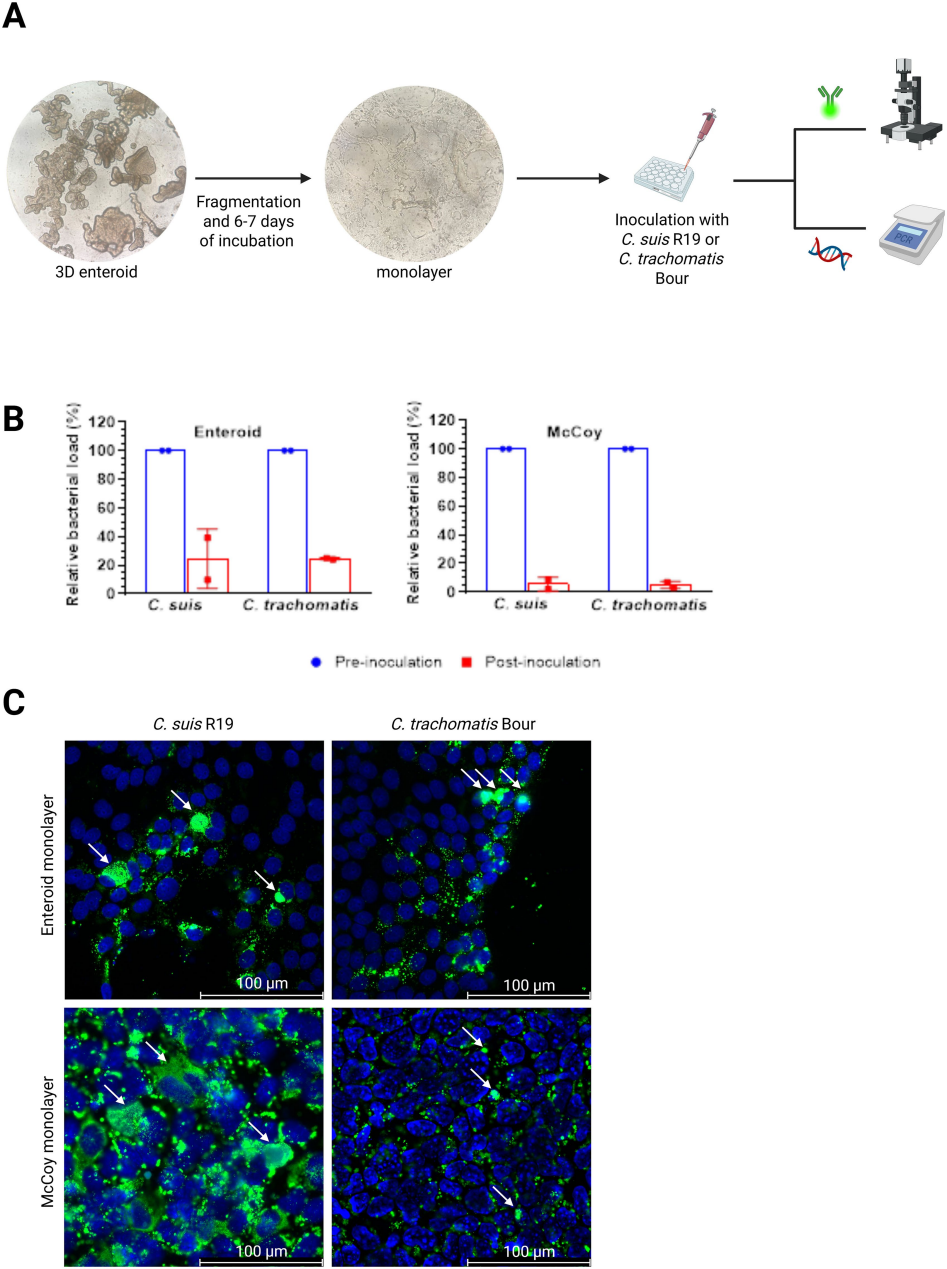
Generation of enteroid-derived 2D monolayers and assessment of chlamydial attachment. **(A)** Schematic overview illustrating the formation of 2D monolayers from 3D enteroids, followed by bacterial inoculation and subsequent analyses, including chlamydial LPS staining and real-time PCR. **(B)** Attachment of *C. suis* R19 and *C. trachomatis* Bour to enteroid and McCoy monolayers, as determined by real-time PCR analysis of the inoculum before and after inoculation following a 1 h contact period. The reduction in chlamydial DNA levels reflects the fraction of bacterial particles that became attached to the epithelial cells, while the remaining signal corresponds to non-attached particles. Data are presented as the mean ± SD (*n* = 2 independent experiments using enteroids derived from two pigs: Supplementary Figure 1), and are expressed relative to the corresponding pre-inoculation condition. **(C)** Immunofluorescence staining of enteroid and McCoy monolayers infected with 10^6^ IFU/ml *C. suis* R19 or 10^6^ IFU/mL *C. trachomatis* Bour. Chlamydial inclusions are shown in green and cell nuclei in blue. Chlamydial inclusions are indicated by arrows. Images are representative of monolayers generated from enteroids derived from a single pig and are representative for monolayers derived from two pigs (see Supplementary Figure 2). Scale bar = 100 μm.

To assess whether *C. suis* is capable to infect primary porcine intestinal epithelial cells, jejunal enteroids were used. As the natural route of intestinal infection occurs via the apical surface of the gut epithelial cells, direct access to the apical surface is required. To this end, porcine apical-out enteroids were generated as described (Chen et al., 2025) and inoculated with *C. suis* R19. Unfortunately, the centrifugation step in the inoculation protocol disrupted the 3D structure of the porcine apical-out enteroids preventing further analysis. We then switched to 2D enteroid monolayers to inoculate primary intestinal epithelial cells via the apical surface with the different *Chlamydia* species and assessed infection by immunofluorescence staining and real-time PCR-based quantification of chlamydial DNA (Figure 1A).

Attachment of *C. suis* R19 and *C. trachomatis* Bour to enteroid or McCoy monolayers was assessed indirectly by real-time PCR by comparing the bacterial load (expressed as IFU/mL values) of the inoculum before and after inoculation. A decreasing trend in bacterial load was observed, indicating attachment of both *Chlamydia* species to enteroid or McCoy monolayers (Figure 1B).

### 3.2 *Chlamydia* infects porcine primary intestinal epithelial cells

To further analyze the presence of *C. suis* and *C. trachomatis* in primary intestinal epithelial cells, enteroid-derived and McCoy monolayers were stained to detect chlamydial LPS 2 days post infection. Clear fluorescent *C. suis* R19 or *C. trachomatis* Bour inclusions were observed in the enteroid monolayers as well as in the reference McCoy monolayers (Figure 1C). The presence of these inclusions indicates successful bacterial entry, intracellular survival and the formation of inclusions in the intestinal epithelial model.

**Figure 2:**
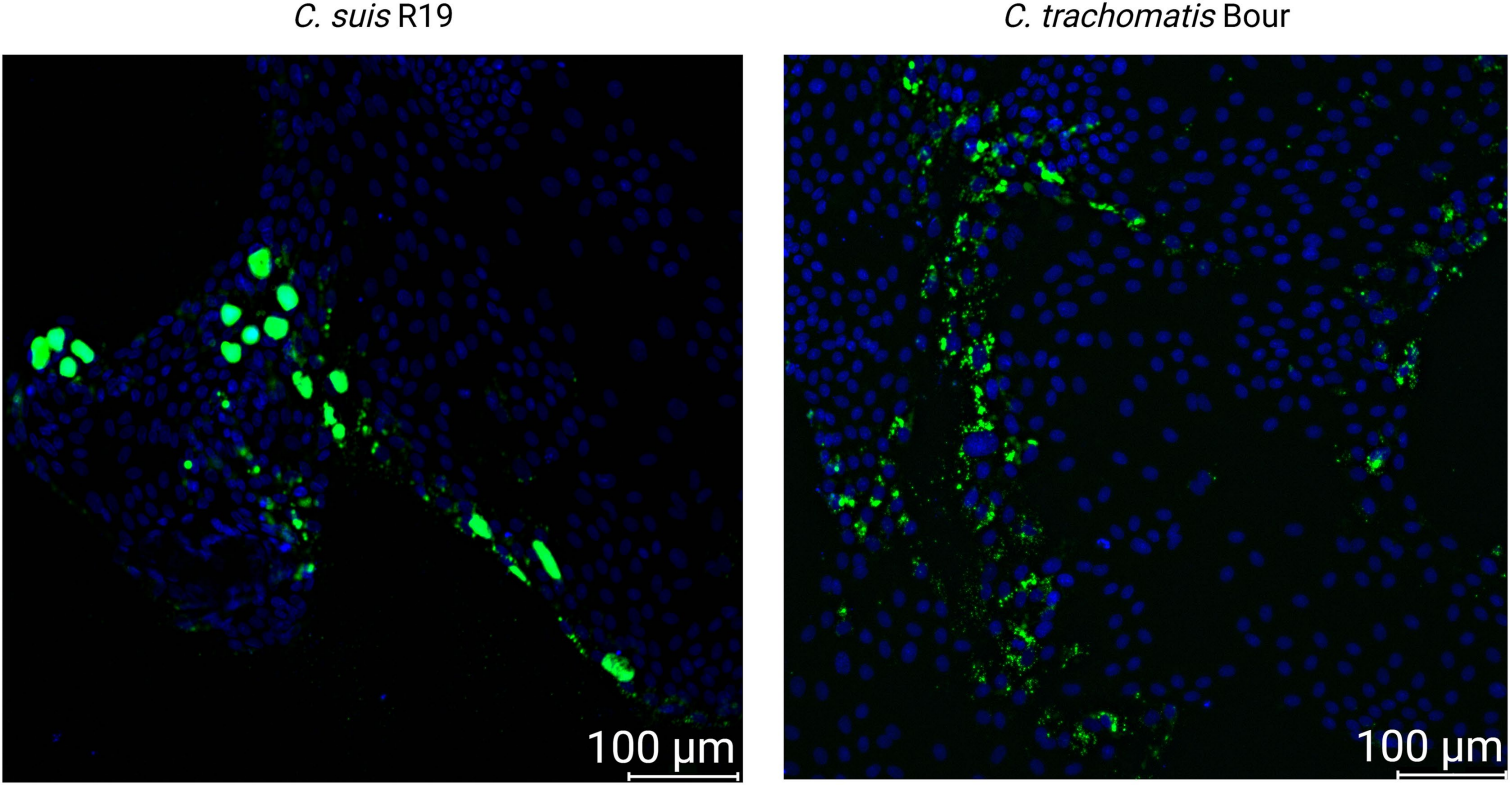
Fluorescence images of enteroid and McCoy monolayers. Immunofluorescence staining of enteroid and McCoy monolayers infected with 10^6^ IFU/ml *C. suis* R19 or 10^6^ IFU/mL *C. trachomatis* Bour. Chlamydial inclusions are shown in green and cell nuclei in blue. Images are representative of monolayers generated from enteroids derived from a single pig and are representative for monolayers derived from two pigs (see Supplementary Figure 2). Scale bar = 100 μm.

Following confirmation of inclusion formation for both *Chlamydia* species, qualitative differences in infection patterns were observed in enteroid-derived monolayers between *C. suis* R19 and *C. trachomatis* Bour. Infection with *C. suis* R19 displayed a more focal and heterogeneous pattern, characterized by clustered inclusions confined to discrete regions of the monolayer, often at the periphery of the epithelial cell clusters. In contrast, *C. trachomatis* exhibited a more homogeneous distribution, with inclusions spread across a larger proportion of epithelial cells, preferentially localized at the periphery of the epithelial cell clusters (Figure 2).

**Figure 3:**
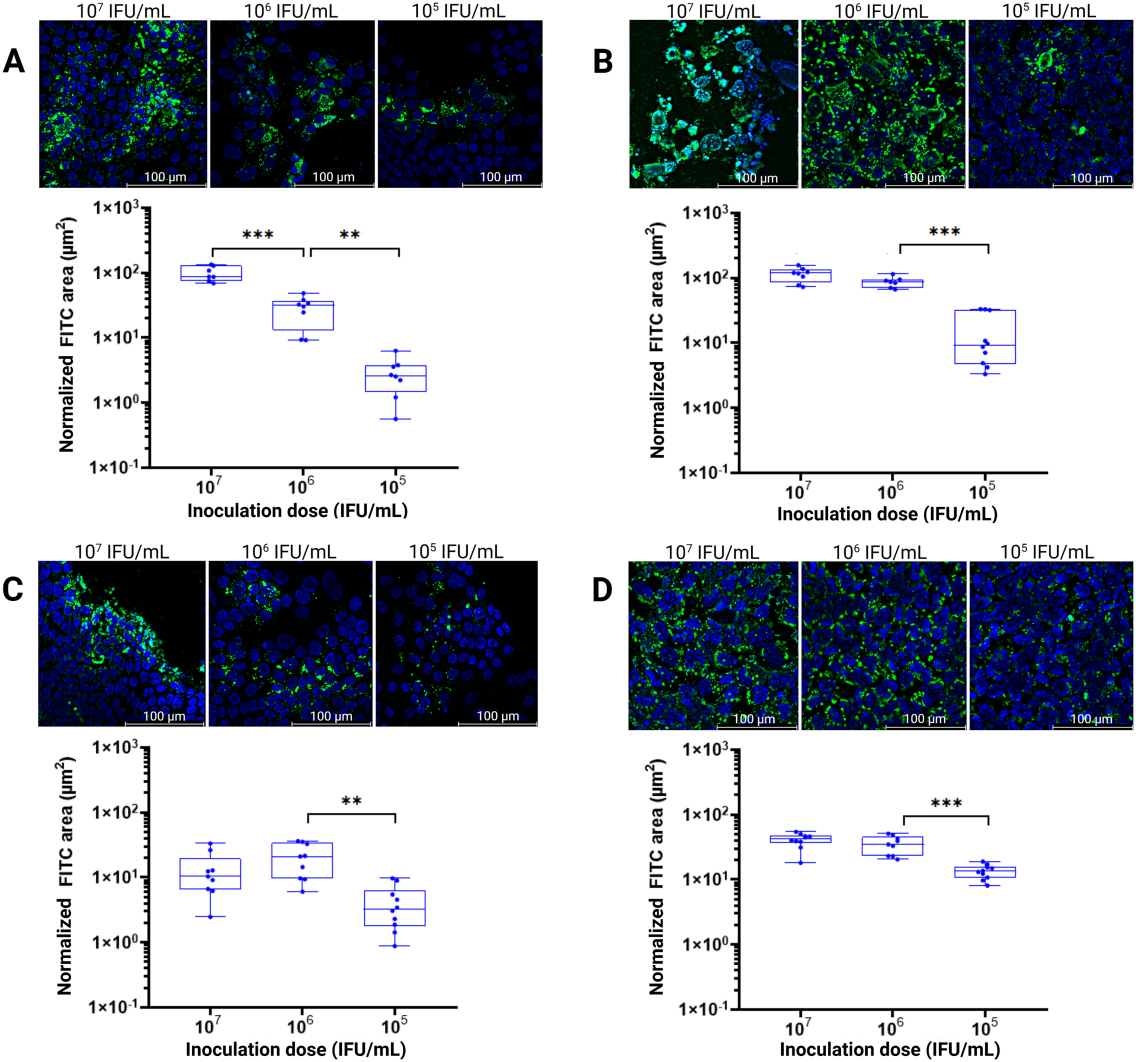
Quantification of infection in enteroid and McCoy monolayers. Fluorescent images of enteroid and McCoy monolayers infected with different bacterial concentrations of *C. suis* R19 (**A,B** resp.) or *C. trachomatis* Bour (**C,D** resp.) at 2 days post-inoculation. The total FITC-positive area (μm^2^) was quantified and normalized to the number of nuclei. Scale bar = 100 μm. Data are presented as the mean ± SD of 10 random fields of one monolayer from one animal. A one-way Welch ANOVA test was performed, followed by Dunnett’s multiple-comparisons post-hoc test to compare the FITC areas across the different inoculation doses. Statistical significance is indicated as follows: *p* < 0.01 (**), and *p* < 0.001 (***).

To quantify infection efficiency across a range of inoculation doses and assess model robustness, dose-dependent infection experiments were performed. Enteroid and McCoy monolayers were inoculated with different bacterial concentrations (10^7^, 10^6^, and 10^5^ IFU/mL) of *C. suis* R19 or *C. trachomatis* Bour. To detect *Chlamydia,* monolayers were stained with LPS-specific monoclonal antibodies, and the total FITC-positive area (μm^2^) was quantified, as the FITC signal corresponds to chlamydial LPS. A clear relationship was observed between the normalized FITC positive area and inoculation dose, with higher doses yielding significantly more FITC-positive areas, indicating consistent performance of the enteroid model across the tested dose range. A similar trend was observed in the reference McCoy host cell line (Figure 3).

**Figure 4:**
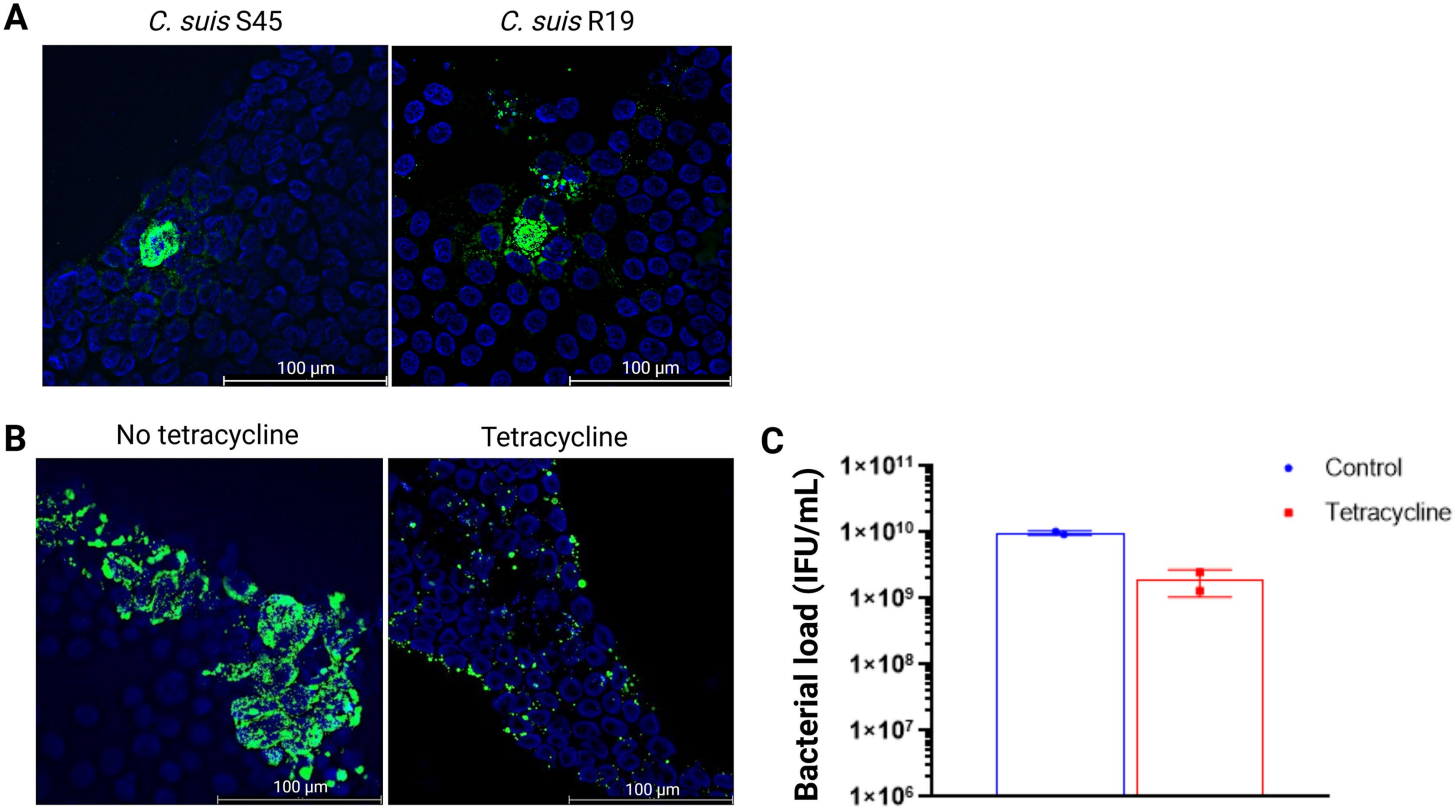
Functional validation of infection. **(A)** Immunofluorescence staining of enteroid monolayers infected with 10^7^ IFU/ml *C. suis* S45 or R19 in the absence of tetracycline. Chlamydial inclusions are shown in green, and cell nuclei are shown in blue. Images are representative of monolayers generated from enteroids derived from a single pig and are representative for monolayers derived from two pigs (see Supplementary Figure 3). Scale bar = 100 μm. **(B)** Immunofluorescence staining of enteroid monolayers infected for 2 days with 10^9^ IFU/mL *C. suis* S45 in the absence (left) and presence (right) of tetracycline. Chlamydial inclusions are shown in green, and cell nuclei are shown in blue. Images are representative of monolayers generated from enteroids derived from a single pig and are representative for monolayers derived from two pigs (see Supplementary Figure 4). Scale bar = 100 μm. **(C)** Bacterial load in the culture medium after 2 days of tetracycline exposure, expressed as IFU/mL. Data are presented as the mean ± SD of two monolayer replicates of one animal.

As antibiotic susceptibility provides a functional readout of active chlamydial infection, we next examined the effect of tetracycline on *C. suis* inclusion formation and extracellular bacterial release in enteroid-derived monolayers. We used both the tetracycline-sensitive reference strain *C. suis* S45 and the tetracycline-resistant strain *C. suis* R19. In the absence of tetracycline, the infection patterns of S45 and R19, each inoculated at 10^7^ IFU/ml, were comparable (Figure 4A). After 2 days of incubation with tetracycline, inclusion numbers of S45 were reduced compared to the control (Figure 4B). Quantification of extracellular bacteria in the culture medium (expressed as IFU/mL) also showed a marked reduction after 2 days of tetracycline exposure (Figure 4B), confirming the inhibitory effect of tetracycline on chlamydial infection.

### 3.3 *Chlamydia* completes its developmental cycle in McCoy cells but not in primary intestinal epithelial cells

To determine whether *Chlamydia* completes its developmental cycle in primary intestinal epithelial cells, the presence of chlamydial DNA in the cells and cell culture medium was evaluated over time using real-time PCR. Sampling time points were selected to correspond to distinct stages of the chlamydial developmental cycle: early entry and EB-to-RB differentiation (2 h), RB replication and inclusion expansion (24 h), release of infectious elementary bodies into the extracellular milieu (48 h), and initiation of a subsequent infection cycle (72 h) (Elwell et al., 2016).

**Figure 5:**
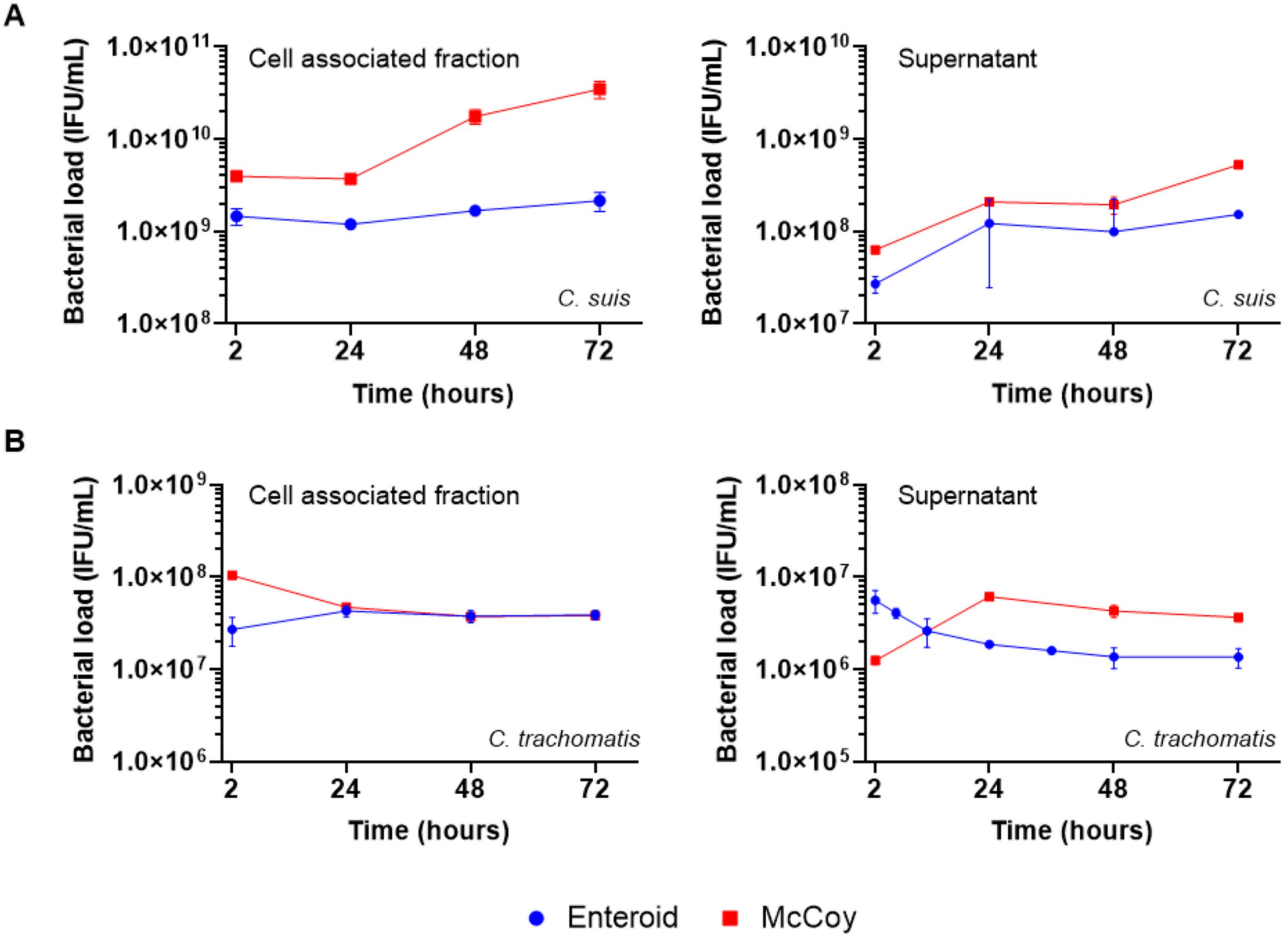
Infection kinetics. Infection of *C. suis* R19 **(A)** and *C. trachomatis* Bour **(B)** in enteroid (blue line, circles) and McCoy monolayers (red line, squares), expressed as IFU/mL based on Ct values obtained by real-time PCR analysis on either the cells or the culture supernatants at different time points post-inoculation. Data are presented as the mean ± SD of two monolayer replicates of one pig (*n* = 1 independent experiment).

After inoculation of enteroid monolayers with 10^7^ IFU/mL *C. suis* R19, no clear increase in cell-associated chlamydial DNA was observed over time, indicating an absence of measurable replication in these cells. In contrast, McCoy cells inoculated with the same infectious dose showed an increase in cell-associated bacterial DNA (Figure 5A). Interestingly, in the culture supernatants of enteroid cultures, extracellular *C. suis* R19 DNA increased over time, similar to the pattern observed in inoculated McCoy cells (Figure 5A). This suggests that bacterial DNA accumulated in the extracellular compartment even in the absence of detectable intracellular replication.

After inoculation of enteroid 2D monolayers with 10^7^ IFU/mL *C. trachomatis* Bour, no increase in cell-associated chlamydial DNA was detected over time, consistent with the pattern observed in McCoy cells inoculated with the same dose (Figure 5B). Similarly, culture supernatants from *C. trachomatis* Bour-infected enteroid monolayers showed no increase in extracellular bacterial DNA over time, in contrast to the increase observed in supernatants from infected McCoy cultures (Figure 5B). These findings indicate that, under the tested conditions, enteroid cultures did not support an increase in either intracellular or extracellular *C. trachomatis* Bour DNA, whereas extracellular bacterial DNA increased at 24 h in the supernatants of infected McCoy cells.

## 4 Discussion

*C. suis* is frequently detected in pigs, and previous studies have indicated that the gastrointestinal tract is an important site of colonization, potentially serving as a niche for infection and persistence (Englund et al., 2012; Yeruva et al., 2013; Dai et al., 2016; Wang et al., 2016; Shao et al., 2018). However, limited information is available on how intestinal colonization influences bacterial persistence, transmission, and reinfection (Zhong, 2021; Tian et al., 2024). This knowledge gap is largely due to the lack of experimental models that accurately mimic the interactions between *C. suis* with the gut epithelium, leaving intestinal chlamydial infections poorly understood. Although recent work has validated human intestinal organoids for modeling enteric *C. trachomatis* infections (Hovhannisyan et al., 2024), in the present study we evaluated a jejunum-derived porcine enteroid model to assess its suitability for studying *C. suis* infection in an intestinal context.

As *Chlamydia* encounters the apical surface of the epithelium during natural infection, experimental access to the apical membrane is required (Hafner et al., 2008; Bishop et al., 2020). Although porcine apical-out enteroids were initially developed for this purpose, technical limitations associated with the inoculation procedure precluded their use. Previous work has shown that porcine crypt-derived 3D intestinal apical-out enteroids can be infected *in vitro*, as demonstrated by successful rotavirus infection (Yan et al., 2023). To date, chlamydial infection of 3D intestinal apical-out enteroids has not been investigated. However, *Chlamydia* infection has been successfully established in murine endometrial 3D organoid models (Bishop et al., 2020), primarily using *C. trachomatis* lymphogranuloma venereum (LGV) strains, which are more invasive and virulent than *C. trachomatis* Bour, as well as *C. suis* R19. These strain-specific differences may partly explain variation in infection efficiency. For these reasons, enteroid-derived 2D monolayers were employed, enabling direct apical infection of primary intestinal epithelial cells (Stroulios et al., 2021). Infection of the apical surface with *C. suis* and *C. trachomatis* was highly efficient, whereas a previous study reported limited apical infectivity in human intestinal enteroids (Hovhannisyan et al., 2024). Possible explanations for this discrepancy include differences in host species, monolayer culture conditions (Millicell® polycarbonate membranes versus collagen IV coating), inoculation procedures related to the *Chlamydia* strains used (absence versus application of centrifugation at 800 × *g* for 60 min), and the epithelial differentiation state. The culture surface can influence epithelial cell differentiation. Polycarbonate membranes, such as Millicell® inserts, lack key physiological features of the extracellular matrix (ECM), whereas collagen IV coatings provide an ECM-associated substrate that supports epithelial cell adhesion, polarization, and differentiation (López García et al., 2025).

The peripheral localization of chlamydial inclusions within epithelial islands, a pattern also reported previously (Hovhannisyan et al., 2024), suggests that local variations in cell density, polarization, or cell type may influence the spatial pattern of infection. In a previous study, SOX9 staining, a marker for progenitor and stem cells, of monolayers derived from fragmented jejunal organoids indicated the presence of stem cells at the periphery of the epithelial islands, which may explain the outward expansion of these islands (Vermeire et al., 2021). Our results suggest that *Chlamydia* preferentially infects cells with stem cell-like characteristics, indicating that these cells may be more permissive to chlamydial infection, although no direct assessment of stem cell markers was performed to confirm their identity. To further assess whether these infected cells retained the capacity for stem cell-driven 3D outgrowth, they were re-cultured as 3D enteroids in ECM material, a process that relies on the ability of stem cells to form enteroid structures. However, this did not result in successful enteroid formation (data not shown), suggesting that infection may impair the capacity for stem cell-driven 3D outgrowth. Previous study using *C. trachomatis* LGV strains in human fallopian tube organoids reported an increase in cells expressing stem cell-associated markers and enhanced organoid-forming capacity (Kessler et al., 2019). These findings suggest that chlamydial infection may influence stem cell-associated pathways, although the outcome may depend on factors such as bacterial strain, inoculum, and tissue-specific properties. Furthermore, successful propagation of *Chlamydia* may also be influenced by the cell cycle state of stem-like cells, as these cells are actively proliferating and give rise to specialized differentiated epithelial cell types (van der Flier and Clevers, 2009). During active proliferation, stem-like cells exhibit increased metabolic activity, providing essential host-derived resources that support intracellular chlamydial replication, and may render these cells a potential reservoir. To date, no studies have specifically investigated whether stem cells can serve as a reservoir for *Chlamydia*. However, as the cellular composition of the enteroid monolayers was not specifically characterized in the present study, it remains unclear whether the observed infection pattern is associated with specific epithelial cell types. The potential role of intestinal stem cells as a reservoir for *Chlamydia* therefore remains a hypothesis and warrants further investigation.

Our results showed that monolayers of primary intestinal epithelial cells permit uptake and inclusion formation of both *C. suis* and *C. trachomatis*. Immunofluorescence staining of *Chlamydia* LPS revealed inclusions within the cells. This phenotype is consistent with observations in other primary epithelial systems, where *C. trachomatis* can form early inclusions in primary human ectocervical epithelial cells, but productive intracellular development and generation of infectious progeny are markedly reduced compared with permissive immortalized HeLa cells (Tang et al., 2021).

In our study, subsequent bacterial proliferation and completion of the developmental cycle appear to be inefficient or aborted, as no productive intracellular replication was detected in these primary cells. As expected, productive infection was detected in McCoy cells. It supports the idea that cell line permissiveness does not necessarily reflect *in vivo* biology and highlights the importance of physiologically relevant models when studying host-pathogen interactions. Several complementary explanations may account for the progressive increase in extracellular *C. suis* DNA in the culture supernatants of enteroid monolayers despite the absence of productive intracellular replication. First, part of the detected DNA may originate from bacteria in the initial inoculum that failed to infect host cells and remained extracellular throughout the experiment. Second, a fraction of bacteria may transiently attach to the epithelial cell surface or initiate invasion inefficiently, followed by release back into the supernatant over time. Third, in the absence of productive intracellular replication, internalized bacteria may undergo degradation or loss of viability, resulting in the gradual accumulation of non-infectious bacterial DNA extracellularly. Another important factor that may contribute to the restricted replication observed in enteroid-derived epithelial cells is the intrinsic innate immune capacity of intestinal epithelial cells. Epithelial cells express a wide range of pattern recognition receptors (PRRs), which recognize pathogen-associated molecular patterns and trigger antimicrobial responses. Activation of these sensing pathways leads to the induction of innate defense mechanisms, including antimicrobial peptide production, autophagy and interferon signaling that limit pathogen survival. In addition to maintaining tissue homeostasis, these responses enable epithelial cells to rapidly respond to infection and prevent intracellular pathogen proliferation (Constant et al., 2021). As primary intestinal epithelial cells retain these physiologically relevant immune functions, they may create a more restrictive intracellular environment compared to immortalized cell lines such as McCoy cells. Consequently, while *Chlamydia* can enter enteroid-derived epithelial cells and initiate inclusion formation, intrinsic epithelial immune defenses may prevent completion of the developmental cycle, resulting in restricted or abortive infection. Future mechanistic studies could help identify host factors restricting chlamydial replication. Cytokine stimulation experiments, for example using interferon-*γ* or other pro-inflammatory mediators, may help clarify the contribution of epithelial innate immune responses to bacterial restriction. Similarly, iron or nutrient supplementation studies could determine whether metabolic limitations within primary intestinal epithelial cells impair RB proliferation or EB differentiation. Finally, transcriptomic and proteomic profiling of infected enteroids would provide valuable insight into host cellular responses and pathways associated with restrictive or permissive infection, thereby improving understanding of host-pathogen interactions in the intestinal epithelium.

In addition, under such stress conditions, *Chlamydia* may enter a persistent state characterized by the formation of enlarged, non-dividing aberrant bodies, which remain viable but are unable to complete the normal developmental cycle, as previously detected in a human enteroid model (Hovhannisyan et al., 2024), and which could be further confirmed at the ultrastructural level by transmission electron microscopy (TEM).

In our study, *C. trachomatis* Bour could not complete its reproduction cycle in primary porcine intestinal epithelial cells. A previous study demonstrated that *C. trachomatis* was able to complete its developmental cycle in human enteroids using the invasive L2 strain at a multiplicity of infection (MOI) of 5 (Hovhannisyan et al., 2024). This discrepancy may be attributed to several experimental differences, including the use of a more invasive strain, a higher infectious dose, and variations in the infection protocol, such as the absence of centrifugation. In addition, the use of a host model, in which a human-adapted pathogen (*C. trachomatis*) was studied in human enteroids, may have further facilitated productive infection. However, neither species replicated efficiently in primary porcine intestinal epithelial cells, suggesting that these cells may constitute a restrictive intracellular environment that limits productive chlamydial replication regardless of species origin.

The precise mechanisms by which intestinal epithelial cells restrict chlamydial replication remain unclear and warrant further investigation. To directly assess whether *Chlamydia* completes its developmental cycle and produces infectious progeny, supernatants and/or cell-associated bacteria from infected enteroid-derived monolayers can be collected and used to inoculate a permissive cell line such as McCoy cells. Observation of inclusion formation in these secondary host cells would provide direct evidence of infectious EB production (Koster et al., 2022). Additionally, TEM can further visualize inclusions and distinguish between RBs, fully differentiated EBs and, if present, aberrant bodies. The presence of fully developed EBs within inclusions would confirm completion of the developmental cycle, as demonstrated in a previous study using human enteroids (Hovhannisyan et al., 2024). In addition, further optimization of the *Chlamydia* inoculation protocol for 3D apical-out enteroids is necessary to determine whether an intact intestinal epithelium could support productive replication and more accurately recapitulate *in vivo* infection dynamics. Such optimization may include improving bacterial access to the apical epithelial surface through microinjection into the enteroid lumen (Bartfeld and Clevers, 2015; Stroulios et al., 2021), increasing inoculation efficiency by prolonged exposure times, and adjusting the multiplicity of infection to account for the 3D architecture.

While this study provides valuable insights into chlamydial infection in primary pig intestinal epithelial cells, several limitations should be acknowledged. The 2D monolayer format does not fully recapitulate the complex 3D architecture and spatial organization of the intestinal epithelium observed *in vivo*, which may affect cellular differentiation, polarity, and host-pathogen interactions. The limited lifespan of primary epithelial monolayers also restricts the duration of experiments and may prevent investigation of long-term infection dynamics. In addition, the establishment and maintenance of enteroid-derived cultures are technically demanding and costly compared to conventional immortalized cell lines, which may limit their scalability for high-throughput applications.

In conclusion, porcine jejunum-derived organoids were used to investigate *C. suis* infection biology infection, comprising bacterial uptake, inclusion formation, and extracellular release, with parallel comparative analyses using *C. trachomatis*. This work extends a previous study that examined enteric *C. trachomatis* infection in human intestinal organoids, which may be subject to ethical and practical constraints, including donor variability and limited accessibility. In this context, pigs represent a relevant alternative model, as their gastrointestinal anatomy, physiology, and immunological features closely resemble those of humans (De Clercq et al., 2020; Tian et al., 2024). Our findings indicate that while initial infection and inclusion formation can occur in these primary intestinal epithelial cells, completion of the chlamydial developmental cycle is restricted. Despite this limitation, the organoid-derived monolayers provide a physiologically relevant *in vitro* platform to study early host-pathogen interactions in the porcine intestine. They offer insights into chlamydial infection dynamics without the need for costly *in vivo* experiments and provide a useful system for exploring the intestinal biology of both zoonotic and human-relevant *Chlamydia* species.

## Data Availability

The original contributions presented in the study are included in the article/Supplementary material, further inquiries can be directed to the corresponding author.

## References

[ref1] Bartfeld S. Clevers H. (2015). Organoids as model for infectious diseases: culture of human and murine stomach organoids and microinjection of *Helicobacter Pylori*. J. Vis. Exp. 2015:e53359. doi: 10.3791/53359, 26650279 PMC4692704

[ref2] Bishop R. C. Boretto M. Rutkowski M. R. Vankelecom H. Derré I. (2020). Murine endometrial organoids to model *Chlamydia* infection. Front. Cell. Infect. Microbiol. 10:416. doi: 10.3389/fcimb.2020.00416, 32923409 PMC7456808

[ref3] Borel N. Regenscheit N. Di Francesco A. Donati M. Markov J. Masserey Y. . (2012). Selection for tetracycline-resistant *Chlamydia suis* in treated pigs. Vet. Microbiol. 156, 143–146. doi: 10.1016/j.vetmic.2011.10.011, 22036200

[ref4] Chen L. Van Der Weken H. Zwaenepoel O. Okkelman I. A. Aelvoet J. Van Denberghe E. . (2025). Cell-specific mRNA delivery via nanobody-functionalized lipid nanoparticles. J. Control. Release 388:114365. doi: 10.1016/j.jconrel.2025.114365, 41161497

[ref5] Constant D. A. Nice T. J. Rauch I. (2021). Innate immune sensing by epithelial barriers. Curr. Opin. Immunol. 73, 1–8. doi: 10.1016/j.coi.2021.07.014, 34392232 PMC8648961

[ref6] Dai J. Zhang T. Wang L. Shao L. Zhu C. Zhang Y. . (2016). Intravenous inoculation with *Chlamydia muridarum* leads to a long-lasting infection restricted to the gastrointestinal tract. Infect. Immun. 84, 2382–2388. doi: 10.1128/IAI.00432-16, 27271744 PMC4962645

[ref7] De Clercq E. Kalmar I. Vanrompay D. (2013). Animal models for studying female genital tract infection with *Chlamydia trachomatis*. Infect. Immun. 81, 3060–3067. doi: 10.1128/IAI.00357-13, 23836817 PMC3754237

[ref8] De Clercq E. Van Gils M. Schautteet K. Devriendt B. Kiekens C. Chiers K. . (2020). *Chlamydia trachomatis* L2c infection in a porcine model produced urogenital pathology and failed to induce protective immune responses against reinfection. Front. Immunol. 11:555305. doi: 10.3389/fimmu.2020.555305, 33193323 PMC7649141

[ref9] De Puysseleyr L. De Puysseleyr K. Braeckman L. Morré S. A. Cox E. Vanrompay D. (2017a). Assessment of *Chlamydia suis* infection in pig farmers. Transbound. Emerg. Dis. 64, 826–833. doi: 10.1111/tbed.12446, 26576707

[ref10] De Puysseleyr K. De Puysseleyr L. Dhondt H. Geens T. Braeckman L. Morré S. A. . (2014a). Evaluation of the presence and zoonotic transmission of *Chlamydia suis* in a pig slaughterhouse. BMC Infect. Dis. 14:560. doi: 10.1186/s12879-014-0560-x, 25358497 PMC4216655

[ref11] De Puysseleyr K. De Puysseleyr L. Geldhof J. Cox E. Vanrompay D. (2014b). Development and validation of a real-time PCR for *Chlamydia suis* diagnosis in swine and humans. PLoS One 9:e96704. doi: 10.1371/journal.pone.0096704, 24816542 PMC4016100

[ref12] De Puysseleyr L. De Puysseleyr K. Rybarczyk J. Donck P. V. De Vos W. H. Vanrompay D. (2021). Transferrins reduce replication of *Chlamydia suis* in McCoy cells. Pathogens 10:858. doi: 10.3390/pathogens10070858, 34358007 PMC8308531

[ref13] De Puysseleyr L. De Puysseleyr K. Vanrompay D. De Vos W. H. (2017b). Quantifying the growth of *Chlamydia suis* in cell culture using high-content microscopy. Microsc. Res. Tech. 80, 350–356. doi: 10.1002/jemt.22799, 27862609

[ref14] Dimond Z. E. Hefty P. S. (2021). Comprehensive genome analysis and comparisons of the swine pathogen, *Chlamydia suis* reveals unique ORFs and candidate host-specificity factors. Pathog. Dis. 79:ftaa035. doi: 10.1093/femspd/ftaa035, 32639528 PMC7948067

[ref15] Donati M. Balboni A. Laroucau K. Aaziz R. Vorimore F. Borel N. . (2016). Tetracycline susceptibility in *Chlamydia suis* pig isolates. PLoS One 11:e0149914. doi: 10.1371/journal.pone.0149914, 26913523 PMC4767523

[ref16] Elwell C. Mirrashidi K. Engel J. (2016). *Chlamydia* cell biology and pathogenesis. Nat. Rev. Microbiol. 14, 385–400. doi: 10.1038/nrmicro.2016.30, 27108705 PMC4886739

[ref17] Englund S. Hård af Segerstad C. Arnlund F. Westergren E. Jacobson M. (2012). The occurrence of *Chlamydia* spp. in pigs with and without clinical disease. BMC Vet. Res. 8:9. doi: 10.1186/1746-6148-8-9, 22280482 PMC3307427

[ref18] Everett K. D. Bush R. M. Andersen A. A. (1999). Emended description of the order *Chlamydiales*, proposal of *Parachlamydiaceae* fam. Nov. and *Simkaniaceae* fam. Nov., each containing one monotypic genus, revised taxonomy of the family *Chlamydiaceae*, including a new genus and five new species, and standards for the identification of organisms. Int. J. Syst. Bacteriol. 49, 415–440. doi: 10.1099/00207713-49-2-415 10319462

[ref19] Guscetti F. Schiller I. Sydler T. Heinen E. Pospischil A. (2009). Experimental enteric infection of gnotobiotic piglets with *Chlamydia suis* strain S45. Vet. Microbiol. 135, 157–168. doi: 10.1016/j.vetmic.2008.09.038, 18950966

[ref20] Hafner L. Beagley K. Timms P. (2008). *Chlamydia trachomatis* infection: host immune responses and potential vaccines. Mucosal Immunol. 1, 116–130. doi: 10.1038/mi.2007.19, 19079169

[ref21] Hoffmann K. Schott F. Donati M. Di Francesco A. Hässig M. Wanninger S. . (2015). Prevalence of chlamydial infections in fattening pigs and their influencing factors. PLoS One 10:e0143576. doi: 10.1371/journal.pone.0143576, 26619187 PMC4664257

[ref22] Hovhannisyan P. Stelzner K. Keicher M. Paprotka K. Neyazi M. Pauzuolis M. . (2024). Infection of human organoids supports an intestinal niche for *Chlamydia trachomatis*. PLoS Pathog. 20:e1012144. doi: 10.1371/journal.ppat.1012144, 39172739 PMC11340892

[ref23] Kessler M. Hoffmann K. Fritsche K. Brinkmann V. Mollenkopf H. J. Thieck O. . (2019). Chronic *Chlamydia* infection in human organoids increases stemness and promotes age-dependent CpG methylation. Nat. Commun. 10:1194. doi: 10.1038/s41467-019-09144-7, 30886143 PMC6423033

[ref24] Koster S. Gurumurthy R. K. Kumar N. Prakash P. G. Dhanraj J. Bayer S. . (2022). Modelling *Chlamydia* and HPV co-infection in patient-derived ectocervix organoids reveals distinct cellular reprogramming. Nat. Commun. 13:1030. doi: 10.1038/s41467-022-28569-1, 35210413 PMC8873204

[ref25] Li L. Fu F. Guo S. Wang H. He X. Xue M. . (2019). Porcine intestinal enteroids: a new model for studying enteric coronavirus porcine epidemic diarrhea virus infection and the host innate response. J. Virol. 93:e01682. doi: 10.1128/JVI.01682-18, 30541861 PMC6384061

[ref26] Li M. Jelocnik M. Yang F. Gong J. Kaltenboeck B. Polkinghorne A. . (2017). Asymptomatic infections with highly polymorphic *Chlamydia suis* are ubiquitous in pigs. BMC Vet. Res. 13:370. doi: 10.1186/s12917-017-1295-x, 29191191 PMC5710075

[ref27] López García F. Leng J. de Oliveira H. Nassoy P. Dementhon K. (2025). Collagen-based gut-on-chip for in vitro modeling of intestinal barrier function and host-pathogen interactions. bioRxiv. doi: 10.1101/2025.05.24.655930

[ref28] Marti H. Kim H. Joseph S. J. Dojiri S. Read T. D. Dean D. (2017). Tet(C) gene transfer between *Chlamydia suis* strains occurs by homologous recombination after co-infection: implications for spread of tetracycline-resistance among Chlamydiaceae. Front. Microbiol. 8:156. doi: 10.3389/fmicb.2017.00156, 28223970 PMC5293829

[ref29] Matsumoto A. Manire G. P. (1970). Electron microscopic observations on the fine structure of cell walls of *Chlamydia psittaci*. J. Bacteriol. 104, 1332–1337. doi: 10.1128/jb.104.3.1332-1337.1970, 16559112 PMC248296

[ref30] Moulder J. W. (1991). Interaction of Chlamydiae and host cells in vitro. Microbiol. Rev. 55, 143–190. doi: 10.1128/mr.55.1.143-190.1991, 2030670 PMC372804

[ref31] Mussard E. Lencina C. Boudry G. Achard C. S. Klotz C. Combes S. . (2023). Culture of piglet intestinal 3D organoids from cryopreserved epithelial crypts and establishment of cell monolayers. J. Vis. Exp. 2023:e64917. doi: 10.3791/64917, 36847381

[ref32] Sachse K. Vretou E. Livingstone M. Borel N. Pospischil A. Longbottom D. (2009). Recent developments in the laboratory diagnosis of chlamydial infections. Vet. Microbiol. 135, 2–21. doi: 10.1016/j.vetmic.2008.09.040, 18986778

[ref33] Schautteet K. de Clercq E. Miry C. van Groenweghe F. Delava P. Kalmar I. . (2013). Tetracycline-resistant *Chlamydia suis* in cases of reproductive failure on Belgian, Cypriote and Israeli pig production farms. J. Med. Microbiol. 62, 331–334. doi: 10.1099/jmm.0.042861-0, 23105027

[ref34] Schiller I. Schifferli A. Gysling P. Pospischil A. (2004). Growth characteristics of porcine chlamydial strains in different cell culture systems and comparison with ovine and avian chlamydial strains. Vet. J. 168, 74–80. doi: 10.1016/S1090-0233(03)00039-X 15158211

[ref35] Shao L. Zhang T. Melero J. Huang Y. Liu Y. Liu Q. . (2018). The genital tract virulence factor pGP3 is essential for *Chlamydia muridarum* colonization in the gastrointestinal tract. Infect. Immun. 86:e00429. doi: 10.1128/IAI.00429-17, 29038127 PMC5736818

[ref36] Stroulios G. Stahl M. Elstone F. Chang W. Louis S. Eaves A. . (2021). Culture methods to study apical-specific interactions using intestinal organoid models. J. Vis. Exp. 2021:e62330. doi: 10.3791/62330 33843928

[ref37] Suchland R. J. Geisler W. M. Stamm W. E. (2003). Methodologies and cell lines used for antimicrobial susceptibility testing of *Chlamydia* spp. Antimicrob. Agents Chemother. 47, 636–642. doi: 10.1128/AAC.47.2.636-642.2003, 12543671 PMC151736

[ref38] Tang C. Liu C. Maffei B. Niragire B. Cohen H. Kane A. . (2021). Primary ectocervical epithelial cells display lower permissivity to *Chlamydia trachomatis* than HeLa cells and a globally higher pro-inflammatory profile. Sci. Rep. 11:5848. doi: 10.1038/s41598-021-85123-7, 33712643 PMC7955086

[ref39] Tian Q. Zhang T. Shu C. Han Z. Huang Y. Wan J. . (2024). Diverse animal models for *Chlamydia* infections: unraveling pathogenesis through the genital and gastrointestinal tracts. Front. Microbiol. 15:1386343. doi: 10.3389/fmicb.2024.1386343, 38605708 PMC11007077

[ref40] van der Flier L. G. Clevers H. (2009). Stem cells, self-renewal, and differentiation in the intestinal epithelium. Annu. Rev. Physiol. 71, 241–260. doi: 10.1146/annurev.physiol.010908.163145, 18808327

[ref41] van der Hee B. Loonen L. M. P. Taverne N. Taverne-Thiele J. J. Smidt H. Wells J. M. (2018). Optimized procedures for generating an enhanced, near physiological 2D culture system from porcine intestinal organoids. Stem Cell Res. 28, 165–171. doi: 10.1016/j.scr.2018.02.013, 29499500

[ref42] Vanrompay D. Ducatellei R. Haesebrouck F. (1992). Diagnosis of avian Chlamydiosis: specificity of the modified Gimenez staining on smears and comparison of the sensitivity of isolation in eggs and three different cell cultures. J. Veterinary Med. Ser. B 39, 105–112. doi: 10.1111/j.1439-0450.1992.tb01144.x, 1621472

[ref43] Verhaeghe M. De Bruyne C. De Meyst A. Rombouts T. Degroote J. Devriendt B. . (2026). Prevalence and tetracycline susceptibility of *Chlamydia suis* in different intestinal sections of pigs from commercial farms. Microorganisms 14:361. doi: 10.3390/microorganisms14020361, 41753648 PMC12942984

[ref44] Vermeire B. Gonzalez L. M. Jansens R. J. J. Cox E. Devriendt B. (2021). Porcine small intestinal organoids as a model to explore ETEC-host interactions in the gut. Vet. Res. 52:94. doi: 10.1186/s13567-021-00961-7, 34174960 PMC8235647

[ref45] Wang L. Zhang Q. Zhang T. Zhang Y. Zhu C. Sun X. . (2016). The *Chlamydia muridarum* organisms fail to auto-inoculate the mouse genital tract after colonization in the gastrointestinal tract for 70 days. PLoS One 11:e0155880. doi: 10.1371/journal.pone.0155880, 27192556 PMC4871562

[ref46] Wanninger S. Donati M. Di Francesco A. Hässig M. Hoffmann K. Seth-Smith H. M. B. . (2016). Selective pressure promotes tetracycline resistance of *Chlamydia suis* in fattening pigs. PLoS One 11:e0166917. doi: 10.1371/journal.pone.0166917, 27893834 PMC5125646

[ref47] Yan M. Su A. Pavasutthipaisit S. Spriewald R. Graßl G. A. Beineke A. . (2023). Infection of porcine enteroids and 2D differentiated intestinal epithelial cells with rotavirus A to study cell tropism and polarized immune response. Emerg. Microbes Infect. 12:2239937. doi: 10.1080/22221751.2023.2239937, 37483148 PMC10399479

[ref48] Yeruva L. Spencer N. Bowlin A. K. Wang Y. Rank R. G. (2013). Chlamydial infection of the gastrointestinal tract: a reservoir for persistent infection. Pathog. Dis. 68, 88–95. doi: 10.1111/2049-632X.12052, 23843274 PMC3751173

[ref49] Zhong G. (2018). *Chlamydia* spreading from the genital tract to the gastrointestinal tract: a two-hit hypothesis. Trends Microbiol. 26, 611–623. doi: 10.1016/j.tim.2017.12.002, 29289422 PMC6003826

[ref50] Zhong G. (2021). *Chlamydia* overcomes multiple gastrointestinal barriers to achieve long-lasting colonization. Trends Microbiol. 29, 1004–1012. doi: 10.1016/j.tim.2021.03.011, 33865675 PMC8510992

