# Supplementary material for: Small intestinal organoids as a model to study interactions of C. suis with its porcine host
Source: Front Microbiol. 2026 Apr 29;17:1818217. doi: 10.3389/fmicb.2026.1818217 (PMC13167934; doi:10.3389/fmicb.2026.1818217)
Supplement: Supplementary file 1 [file Supplementary_file_1.docx]

Supplementary Material

# Supplementary Figures

**Supplementary Figure 1**. **Assessment of chlamydial attachment.** Attachment of *C. suis* R19 and *C. trachomatis* Bour to enteroid and McCoy monolayers, as determined by real-time PCR analysis of the inoculum, pre- and post-inoculation and expressed in IFU/mL. Data are presented as the mean $\pm$ SD of two monolayers of one pig (*n* = 1 independent experiment). **(A)** and **(B)** represent independent experimental repeats in enteroids derived from two pigs.


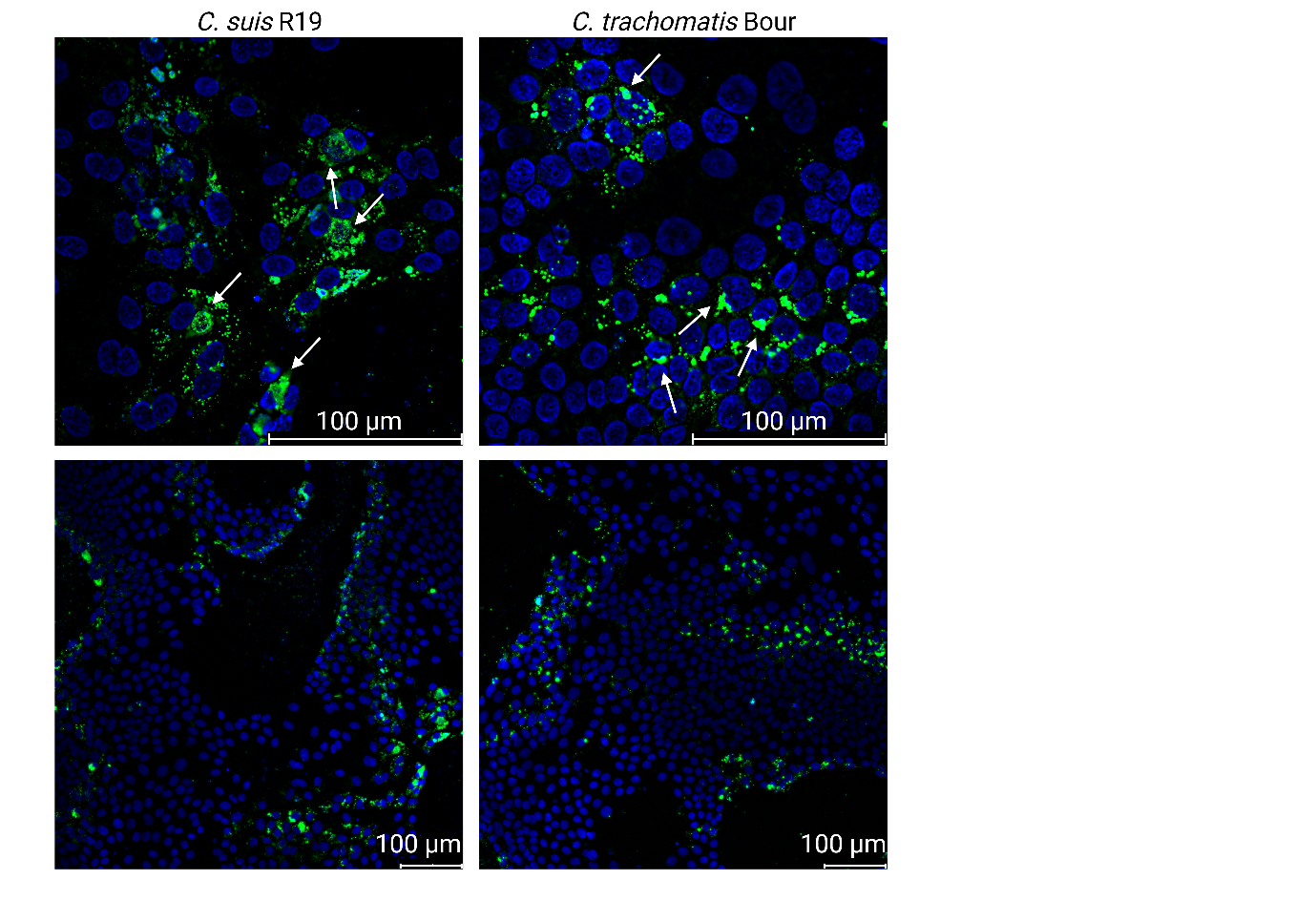


**Supplementary Figure 2**. **Fluorescence images of enteroid and McCoy monolayers.** Immunofluorescence staining of enteroid monolayers from another pig infected with 10^6^ IFU/ml *C. suis* R19 or *C. trachomatis* Bour. Chlamydial inclusions (green) are indicated with an arrow, and cell nuclei are shown in blue. Images are representative of monolayers generated from enteroids derived from a single pig. The lower panels illustrate the distribution of chlamydial inclusions within the enteroid monolayer. Scale bar = 100 µm.

**
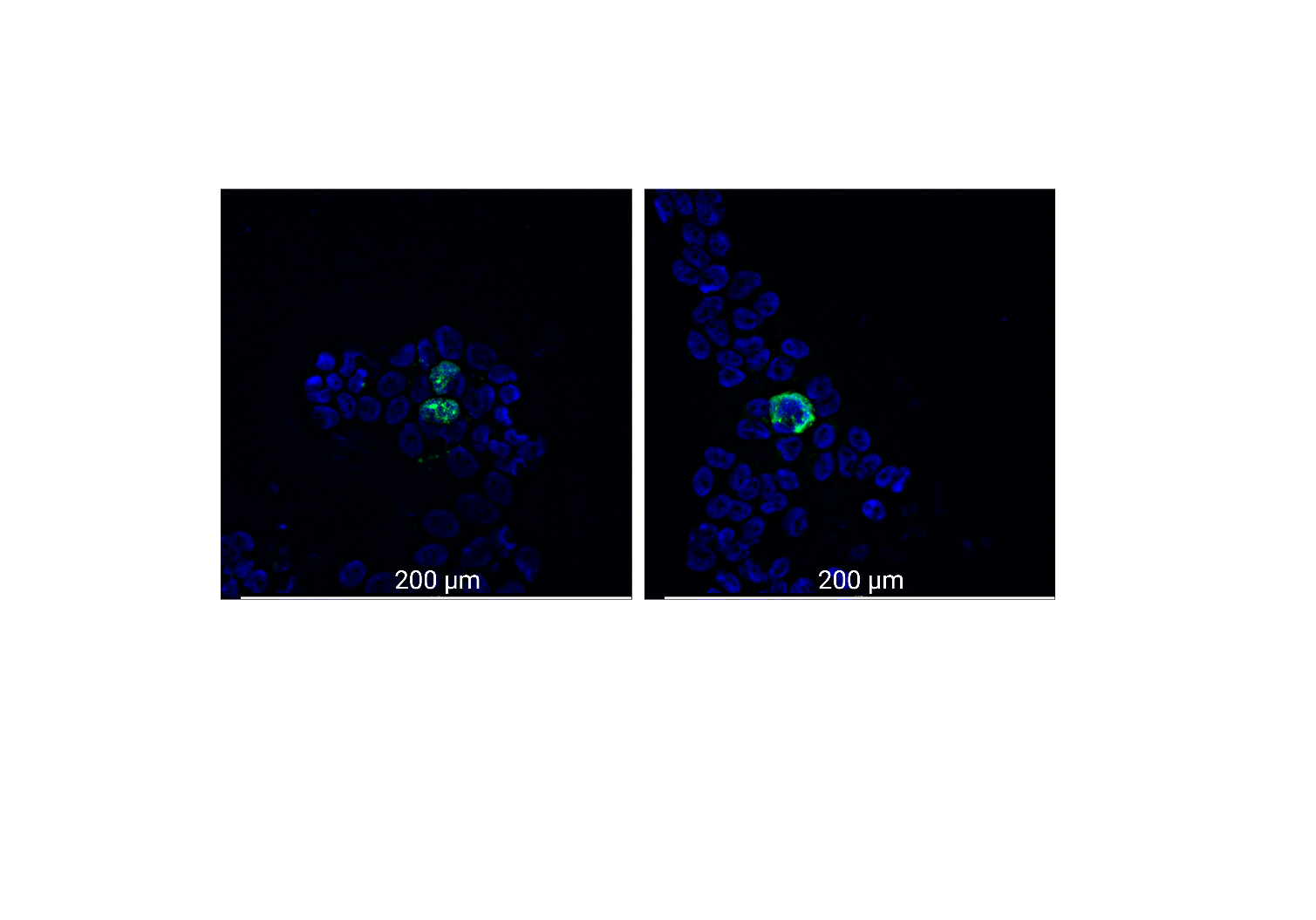
**

**Supplementary Figure 3**. **Fluorescence images of enteroid monolayers.** Immunofluorescence staining of enteroid monolayers from another pig infected for 2 days with 10^7^ IFU/ml *C. suis* S45. Chlamydial inclusions are shown in green, and cell nuclei are shown in blue. Scale bar = 200 µm*.*


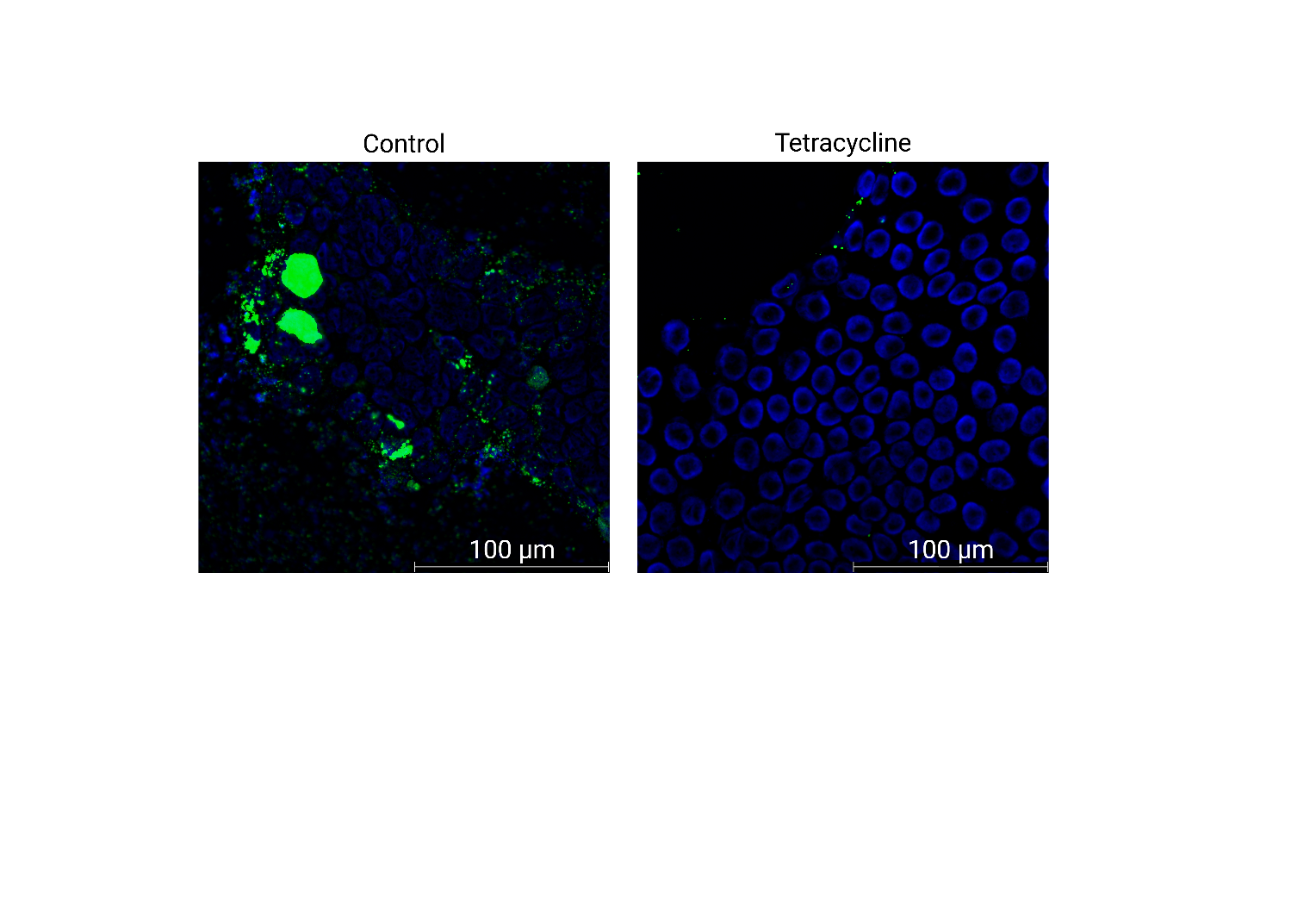

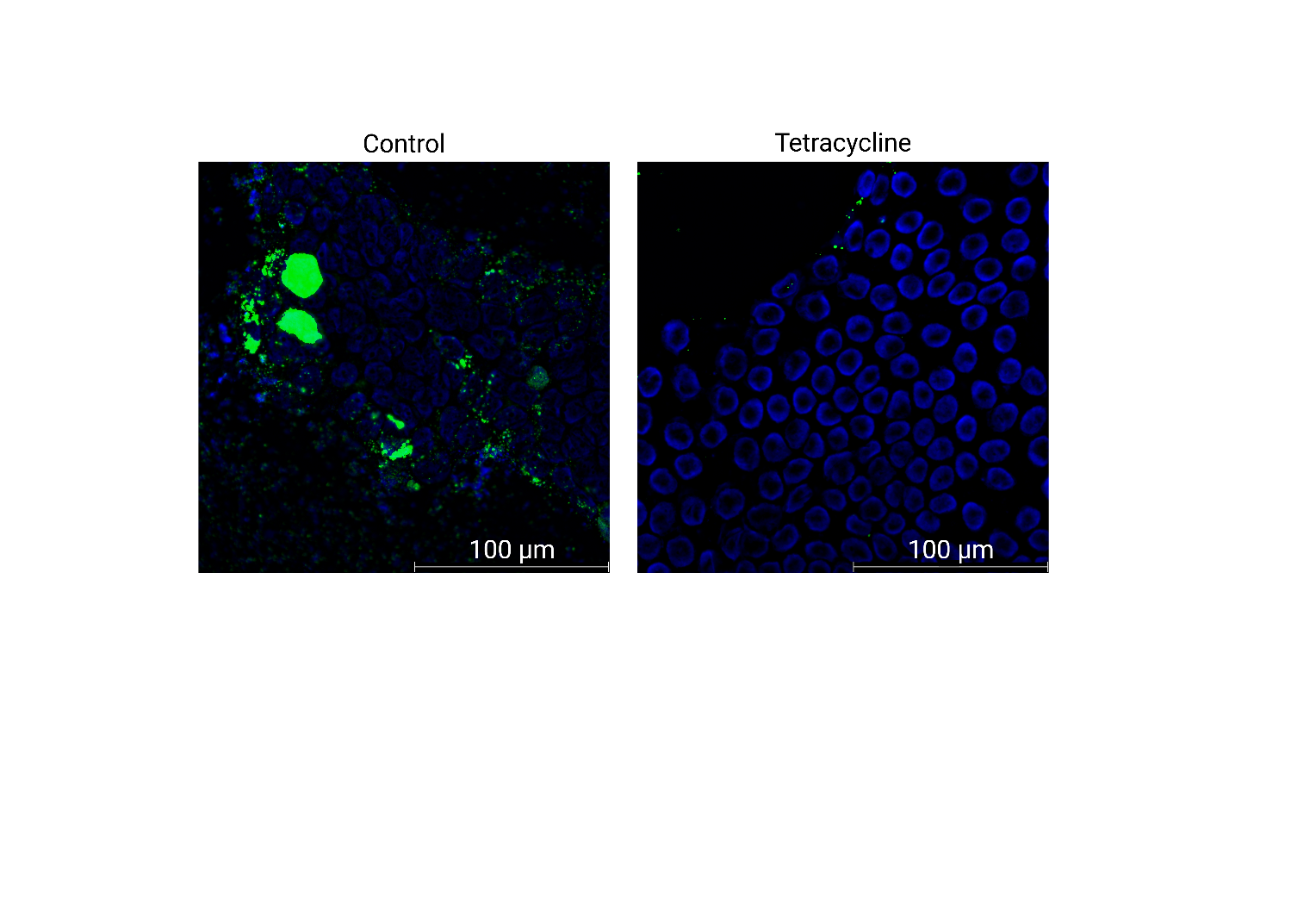


**Supplementary Figure 4.** **Functional validation of infection*.*** Immunofluorescence staining of enteroid monolayers from one other pig infected for 2 days with 10^9^ IFU/mL *C. suis* S45 in the absence (left) and presence (right) of tetracycline. Chlamydial inclusions are shown in green, and cell nuclei are shown in blue. Scale bar = 100 µm.
